# Alternate therapeutic pathways for PARP inhibitors and potential mechanisms of resistance

**DOI:** 10.1038/s12276-021-00557-3

**Published:** 2021-01-25

**Authors:** Dae-Seok Kim, Cristel V. Camacho, W. Lee Kraus

**Affiliations:** 1 Laboratory of Signaling and Gene Regulation, Cecil H. and Ida Green Center for Reproductive Biology Sciences, University of Texas Southwestern Medical Center, Dallas, TX 75390 USA; 2 Division of Basic Research, Department of Obstetrics and Gynecology, University of Texas Southwestern Medical Center, Dallas, TX 75390 USA; 3 Present Address: Touchstone Diabetes Center, Department of Internal Medicine, University of Texas Southwestern Medical Center, Dallas, TX 75390 USA

**Keywords:** Cancer therapy, Mechanisms of disease, PolyADP-ribosylation

## Abstract

Homologous recombination (HR) repair deficiency impairs the proper maintenance of genomic stability, thus rendering cancer cells vulnerable to loss or inhibition of DNA repair proteins, such as poly(ADP-ribose) polymerase-1 (PARP-1). Inhibitors of nuclear PARPs are effective therapeutics for a number of different types of cancers. Here we review key concepts and current progress on the therapeutic use of PARP inhibitors (PARPi). PARPi selectively induce synthetic lethality in cancer cells with homologous recombination deficiencies (HRDs), the most notable being cancer cells harboring mutations in the *BRCA1* and *BRCA2* genes. Recent clinical evidence, however, shows that PARPi can be effective as cancer therapeutics regardless of *BRCA1/2* or HRD status, suggesting that a broader population of patients might benefit from PARPi therapy. Currently, four PARPi have been approved by the Food and Drug Administration (FDA) for the treatment of advanced ovarian and breast cancer with deleterious *BRCA* mutations. Although PARPi have been shown to improve progression-free survival, cancer cells inevitably develop resistance, which poses a significant obstacle to the prolonged use of PARP inhibitors. For example, somatic *BRCA1/2* reversion mutations are often identified in patients with *BRCA1/2*-mutated cancers after treatment with platinum-based therapy, causing restoration of HR capacity and thus conferring PARPi resistance. Accordingly, PARPi have been studied in combination with other targeted therapies to overcome PARPi resistance, enhance PARPi efficacy, and sensitize tumors to PARP inhibition. Moreover, multiple clinical trials are now actively underway to evaluate novel combinations of PARPi with other anticancer therapies for the treatment of PARPi-resistant cancer. In this review, we highlight the mechanisms of action of PARP inhibitors with or without *BRCA1/2* defects and provide an overview of the ongoing clinical trials of PARPi. We also review the current progress on PARPi-based combination strategies and PARP inhibitor resistance.

## Cancer therapy: Expanding the use of a class of drugs that promote DNA damage

PARP inhibitors (PARPi) target a protein called PARP-1 to treat specific types of breast and ovarian cancers with mutations in the *BRCA1* and *BRCA2* genes, but they may be effective against even more cancers. W. Lee Kraus at the University of Texas Southwestern Medical Center, Dallas, USA, and co-workers review how PARPi work, including mechanisms of action, ongoing clincal trials, and combination therapies. They report that PARPi, acting through PARP-1, function by inhibiting DNA repair and causing replication stress, thereby promoting DNA damage and marking cancer cells for destruction. However, cancers develop resistance to PARPi; further research is needed to determine how these drugs can be used to optimize their efficacy. Improving our understanding of how PARPi function could help to guide wider application of these powerful cancer therapeutics.

## Introduction

Poly(ADP-ribose) polymerase-1 (PARP-1) is a ubiquitous nuclear enzyme that utilizes nicotinamide adenine dinucleotide (NAD^+^) to catalyze the addition of ADP-ribose (ADPR) moieties to specific amino acids of target proteins^1^. PARP-1 is the founding and most abundant member of the PARP family of ADP-ribosyl transferases and is responsible for ~80–90% of the polyADPRylation (PARylation) activity in cells^2^. A number of studies have shown that PARP-1 is significantly upregulated in various cancer cell lines and malignant tissues isolated from patients^3,4^. Therefore, PARP-1 has garnered significant attention as a therapeutic target. Currently, four PARP inhibitors (PARPi) primarily targeting PARP-1 have been approved for the treatment of cancers in various settings by the US Food and Drug Administration (FDA). PARPi are thought to act by inhibiting DNA repair and replication in cancer cells deficient in BRCA1/2-dependent homologous recombination (HR) pathways through a process known as synthetic lethality^5^.

Since the discovery of PARPi-induced synthetic lethality in *BRCA1-* or *BRCA2*-deficient cancer cells, a series of studies have elucidated the molecular mechanisms that underlie the action of PARPi. In particular, one major effect of PARPi has been proposed to occur via “trapping” of the PARP-1 protein at sites of DNA damage^6,7^. In addition to blocking PARylation reactions, PARP-1 trapping leads to the establishment of a stable interaction between PARP-1 and genomic DNA in chromatin. These PARPi–PARP-1–DNA complexes interfere with DNA replication by destabilizing replication forks, leading to subsequent instability and cell death^8,9^. Based on the synthetic lethality observed and the “trapping” mechanism of action of PARPi, preclinical and clinical studies have established that patients carrying *BRCA1/2* mutations derive the greatest clinical benefit from PARPi therapy^10–12^. Thus, the FDA has approved several PARPi for the treatment of cancers with deleterious *BRCA1/2* mutations^13,14^.

Recently, multiple clinical and mechanistic studies with PARPi have also highlighted therapeutic responses irrespective of *BRCA1/2* status or HR-mediated DNA repair deficiency^11,15–17^. This new evidence strengthens the rationale for extending the clinical use of PARPi beyond targeting DNA repair toward a wider group of patients, in particular those with a wild-type *BRCA1/2* status. Although PARPi have been shown to effectively increase progression-free survival (PFS) and overall survival in a broad population, the development of resistance to PARPi poses a significant obstacle to their prolonged use^18–20^. This review highlights the current understanding of the mechanism of action of PARPi, describes clinically approved PARPi as maintenance monotherapies or combination therapies for the treatment of cancer, and summarizes several molecular mechanisms of PARPi resistance.

## Key milestones in the molecular characterization of PARPs

**Fig. 1:**
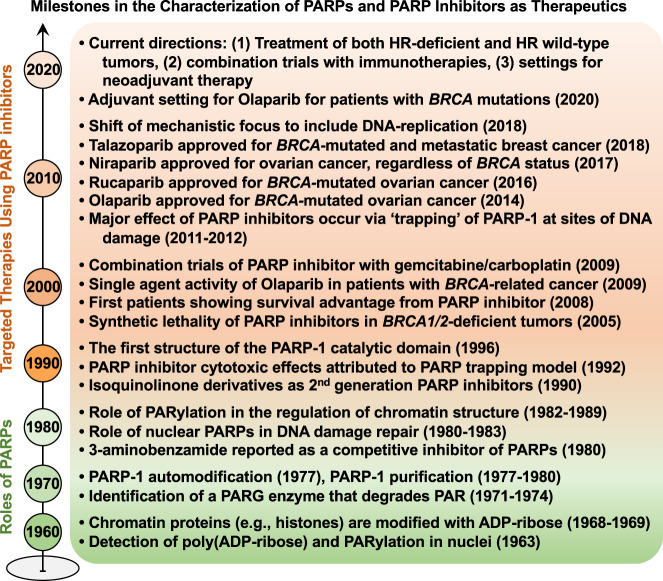
Timeline of major milestones in the characterization of PARPs and PARP inhibitors. Since the initial discovery of poly(ADP-ribose) and PARylation in early 1960, the major molecular mechanisms of PARPs and emergence of PARPi as therapeutics are highlighted by two different colors.

Poly(ADP-ribose) (PAR), synthesized by PARP-1, was first discovered through nicotinamide mononucleotide-induced incorporation of α^32^P-ATP in nuclear extracts (Fig. 1)^21^. Since the discovery of the PARylation reaction in the early 1960s, PARP-1 has been the primary focus of studies on PARPs and ADP-ribosylation^2^. Observations from further biochemical and molecular studies in the 1970s–1990s established that (1) PARP-1 can PARylate itself (automodification)^22–24^, (2) PAR glycohydrolase (PARG) is an abundant enzyme that degrades most of the PAR polymer in cells^25^, (3) histones and other chromatin-associated proteins are modified with ADPR^26,27^, (4) nuclear PARPs play a key role in DNA damage repair^28^, (5) PARP “trapping” may account for the cytotoxic effects of PARPi^29^, and (6) the PARP-1 catalytic domain contains a number of key NAD^+^-binding and catalytic residues, as determined by X-ray crystallography and biochemical analyses^30^ (Fig. 1).

From the late 1990s through the early 2000s, the PARP and ADP-ribosylation field transitioned from validating the biochemical and molecular functions of PARP-1 to exploring the physiological and pathological implications of PARP inhibition using PARPi^5,31^. In particular, growing evidence suggests that dysregulation of PARPs and ADP-ribosylation impacts the biology of various human cancers^3,4,32^. For example, the expression level of various PARPs and their enzymatic activity have been shown to be significantly increased in invasive cancer cell lines^4^, as well as in malignant tissues isolated from patients with cancer^3^. Therefore, given their key roles in various cancers, PARPs are attractive therapeutic targets. As such, the development of PARPi and their clinical applications have attracted considerable interest and opened a promising avenue of research that has the potential to dramatically impact the treatment of diverse cancers. Remarkably, several landmark studies in the mid-2000s showed that PARPi promote synthetic lethality in *BRCA1/2*-mutated cancers and provide a therapeutic benefit to patients with germline or somatic *BRCA1/2* mutations^5^. After more than a decade of research on PARPi, they remain a promising avenue for cancer therapeutics. The development, validation, and FDA approval of PARPi from the 2000s to present are explored in more detail below.

## Emergence of PARPi as therapeutics promoting synthetic lethality

### PARP-1 and SSB repair

**Fig. 2:**
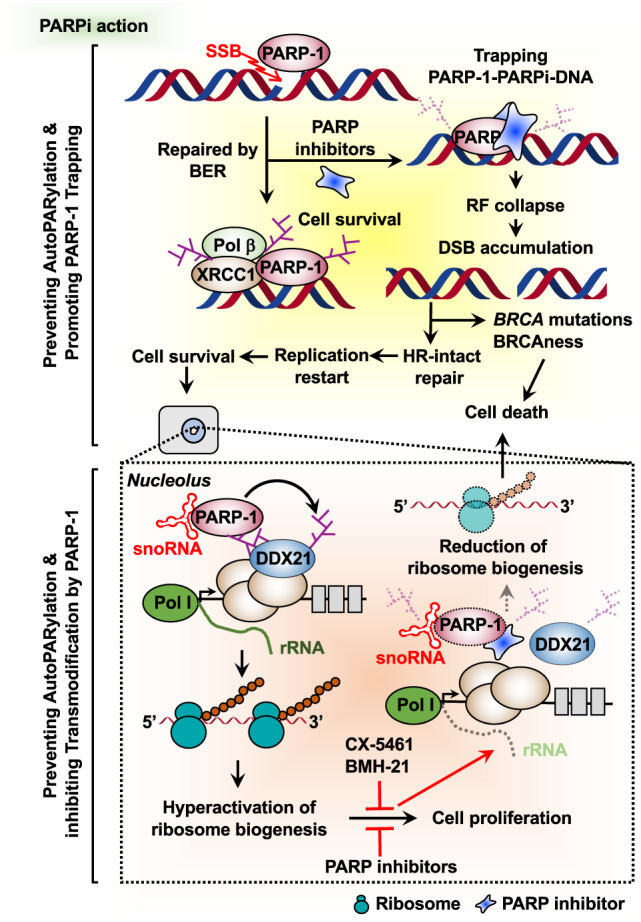
Mechanisms of action of PARP inhibitors in HR-proficient or HR-deficient cancers. PARP-1 activation upon DNA single-strand breaks (SSBs) catalyzes PARylation, which is required for the accumulation and stabilization of base excision repair (BER) components. PARPi selectively induces synthetic lethality by blocking the repair of damaged DNA in the context of cells with homologous recombination (HR) deficiency (upper panel). Alternatively, PARPi reduce hyperactivated ribosome biogenesis by snoRNA-activated PARP-1, regardless of HR-mediated DNA repair deficiency (lower panel).

PARP-1, the most studied PARP family member, is a critical sensor and signal transducer of DNA single-strand breaks (SSBs)^29^. One aspect of PARP-1 function is as a DNA repair enzyme that promotes SSB repair through the base excision repair pathway (Fig. 2, upper left panel)^33^. PARP-1 rapidly detects and binds to DNA at the sites of SSBs, which stimulates PARP-1 activation through an allosteric change in the structure of PARP-1^34^. PARP-1 activation catalyzes a series of PARylation events that promote recruitment of various components of the repair machinery to SSB sites. X-ray repair cross-complementing protein 1, an essential factor in SSB repair, acts as a scaffold for the recruitment, stabilization, and stimulation of multiple enzymatic components of the SSB repair process^33^. In repair-proficient cells, PARP-1 is eventually released from repaired DNA through autoPARylation.

### PARP-1, HR, and replication

HR plays a pivotal role in maintaining genome stability by promoting accurate repair of double-strand breaks (DSBs) and restarting stalled/collapsed replication forks^35^. BRCA1 and BRCA2 have distinct roles in HR-mediated DSB repair and are required for the protection of stalled/collapsed replication forks caused by replication stress^36^. HR-mediated DNA repair can resolve replication-induced DSBs, maintaining the integrity of the genome, restarting replication, and promoting cell survival^36^. However, germline or somatic inactivating mutations in *BRCA1/2* (or other HR factors) render cells exquisitely sensitive to PARP inhibition by leading to the accumulation of toxic DSBs, genomic instability, subsequent cell cycle arrest, and cell death (Fig. 2, upper right panel). Indeed, preclinical and clinical studies of PARPi have confirmed that patients harboring *BRCA1/2* mutations derive the greatest clinical benefit from PARPi treatment^31^. Aside from DNA damage, there is growing evidence that PARPi promote cytotoxic effects by perturbating DNA replication. PARPi are known to interfere with DNA replication by destabilizing replication forks, leading to subsequent cell death^37,38^. In addition, Maya-Mendoza et al.^39^ proposed that PARPi increase replication fork speed, leading to DNA replication stress and cell death.

### PARP-1 trapping

The PARP-1 catalytic domain has a number of key NAD^+^-binding and catalytic residues. PARPi compete with NAD^+^ for binding to the PARP-1 catalytic domain, leading to inhibition of its enzymatic activity and impairing proper DNA repair^34^. PARPi also inhibit PARP-1’s ability to dissociate from damaged DNA, thereby establishing a stable complex of PARP-1 with DNA in a process called PARP trapping^6,34,40^. This PARPi–PARP-1–DNA complex at SSB lesions disrupts the catalytic cycle of PARP-1 in DNA damage signaling by preventing autoPARylation and PARP-1 release from the site of damage. Trapped PARPi–PARP-1–DNA complexes subsequently lead to the accumulation of unrepaired SSBs, which impair proper progression of replication forks and ultimately result in the formation of DNA DSBs (Fig. 2, upper right panel)^6,41^. Mechanistically, the definition of PARP-1 trapping is imprecise, as are the centrifugation-based chromatin retention assays used to measure it. Moreover, the relationships between PARP-1 trapping and the efficacy of PARPi are not always straightforward (see below).

Recent studies using hydrogen/deuterium exchange mass spectrometry (HXMS) combined with X-ray structures have dissected allosteric PARP-1 retention at DNA breaks by different PARPi^40^. HXMS experiments have revealed that an essential allosteric regulatory domain of PARP-1 [i.e., the helical domain (HD)] is stabilized or destabilized by distinct PARPi engaged in the NAD^+^-binding site, resulting in different PARP-1 affinities for DNA breaks^40^. Thus, different PARPi can drive diverse allosteric changes in PARP-1 that modulate PARP-1 trapping capability, ultimately impacting PARPi potency toward cancer cell killing. Indeed, structural modifications of veliparib, which favor PARP-1 release by stabilizing the HD, convert it to an allosteric pro-retention compound with increased trapping ability and cytotoxicity (UKTT15)^40^. In the PARP-1 trapping model, the allosteric effect of different PARPi is only beginning to be understood and further study is needed.

### Synthetic lethality

PARPi are thought to act by blocking DNA repair and replication in cancer cells deficient in HR and HR-mediated DNA repair^5,41^. HR repair deficiency impairs the proper maintenance of genomic stability, thus rendering cancer cells vulnerable to loss or inhibition of DNA repair proteins, such as PARP-1. Thus, the efficacy of PARPi is broadly based on the genetic concept of synthetic lethality, whereby concurrent inhibition of PARP enzymes in cells with an underlying HDR (homology directed repair) defect, particularly *BRCA1* and *BRCA2* mutations, results in cell death. A singular loss of function of either of these alone, however, is still compatible with cell viability (Fig. 2, top panel)^5^. The use of PARP inhibitors exemplifies synthetic lethality in the clinic and the following sections provide an overview of ongoing clinical trials of PARPi^5,34^.

## Expanded application of PARPi in HR-proficient cancers

Historically, studies of PARP-1 have focused on its role in DNA damage repair and genome maintenance, especially with respect to the use of PARPi in cancer treatment, which were initially tested in ovarian cancer patients with *BRCA1* and *BRCA2* mutations^31,34^. However, during the past two decades, the field of PARP-1 and ADP-ribosylation has expanded to include the regulation of chromatin structure, gene expression, RNA processing, ribosome biogenesis, and translation in cancer^1^. These new findings on the diverse and fundamental roles of PARP-1 and ADP-ribosylation may be as important as its effects on DNA repair.

Intriguingly, recent studies have reported that PARPi can confer clinical benefits in patients regardless of *BRCA1/2* or HR-mediated DNA repair status^15^. For example, niraparib has been shown to significantly improve clinical outcomes in patients lacking *BRCA1/2* mutations or HR deficiency (HRD)^11,15^. In addition, PARPi have been shown to be effective at killing cells with intact *BRCA1/2* through non-DNA repair pathways, a role that is not limited to a particular genetic background or type of cancer^16^. A recent study by Keung et al.^17^ surveyed the response to 13 different PARPi in 12 breast cancer cell lines and detected significant sensitivity regardless of *BRCA1/2* status. These findings indicate a broader utility for PARPi in the treatment of cancer patients carrying wild-type *BRCA1/2*. They also provide evidence to suggest that the roles of PARP-1 in DNA damage repair and genetically defective tumors may not be the only basis for its positive therapeutic action.

*BRCA1/2* and HRD status are not the only biomarkers of response to PARPi, and additional biomarkers are necessary to better predict response. Recent studies have identified potential biomarkers related to rDNA transcription by Pol I and ribosome biogenesis. In this regard, cancer cells use various strategies to increase ribosome biogenesis by upregulating rRNA production, which can promote the uncontrolled growth and proliferation of cancer cells^42^. The multiple pathways leading to hyperactivation of Pol I-dependent transcription suggest that cancer cells may be addicted to elevated rRNA synthesis and, therefore, are particularly vulnerable to its inhibition. Recently, inhibiting rDNA transcription using small molecule inhibitors has been considered a therapeutic approach in cancer treatment^43,44^ and several Pol I inhibitors that suppress rDNA transcription are known to have wide-ranging and potent antitumorigenic activity (Fig. 2, lower right panel). Small molecule inhibitors, such as quarfloxin and CX-5461, inhibit rDNA transcription by selectively disrupting nucleolin/rDNA G-quadruplex complexes^43^. In addition, a cell-based, high-throughput screen of synthetic chemical libraries showed that the small molecule BMH-21 binds to ribosomal DNA and represses rDNA transcription in NCI60 cancer cells, limiting tumor growth^44^. Collectively, these results highlight the expanding role of Pol I inhibitors as tractable targets for cancer therapeutics.

In the same vein, a recent study from our laboratory identified an alternate molecular pathway for targeting cancer-enhanced ribosome biogenesis in *BRCA1/2* wild-type breast cancers with PARPi (Fig. 2, lower left panel). Our study identified endogenous snoRNAs as potent activators of PARP-1 enzymatic activity within the nucleolus, leading to PARylation of DDX21, an RNA helicase required for rDNA transcription^16,45^. PARylation of DDX21 promotes ribosome biogenesis by retaining DDX21 in the nucleolus and driving rDNA transcription, ultimately leading to increased cell proliferation. Treatment with PARPi reduces ribosome biogenesis and cell growth through impaired DDX21 nucleolar localization. Taken together, these clinical and mechanistic studies strengthen the rationale for advancing the use of PARPi in clinical trials for cancers with wild-type *BRCA1/2*.

## FDA-approved PARPi

The development and use of PARPi in the clinic provided the first example of therapeutic synthetic lethality in oncology^5,46^. At the time of this review, the ClinicalTrials.gov database contained 110 PARP-related clinical trials completed over the last 10 years (2011–present), as identified using the search terms “cancer” and “PARP.” The database also listed 103 active trials and 141 trials currently in the recruitment phase. To date, four different PARPi have been approved by the FDA: olaparib (2014; Lynparza, AstraZeneca), rucaparib (2016; Rubraca, Clovis Oncology, Inc.), niraparib (2017; Zejula, Tesaro, Inc.), and talazoparib (2018; Talzenna, Pfizer). Each has specific indications for the treatment of ovarian, fallopian tube, breast, and peritoneal cancers (Table 1). The clinical data available indicate that these PARPi can significantly improve PFS. Based on a number of key studies, olaparib was the first PARPi approved for the treatment of advanced ovarian cancers associated with defective *BRCA1/2*, followed by rucaparib, niraparib, and talazoparib for the same cancer types and mutation profile^14^. Many more inhibitors, not discussed herein, are in various stages of development and preclinical testing. The development and evaluation of PARPi, including combination trials with targeted therapy and neoadjuvant setting, are explored in greater detail below.

**Table 1:** Selected features of FDA-approved PARPi.

Drug	Approved	PARP-1 selectivity	Trapping ability	Off-target kinases (computational)^a^	Off-target kinases (CIA)^a^
Olaparib	December 2014	++	+	23	–
Rucaparib	December 2016	+	+	22	CDK16, PIM3, DYRK1B
Niraparib	March 2017	++	++	11	DYRK1A, DYRK1B
Talazoparib	October 2018	+	+++	2	–

*CIA,* catalytic inhibition assays.

Selected features of the four FDA-approved PARPi currently being used in the clinic.

^a^Summarized from Antolin et al.^43^.

### Not all PARPi are created equal

The ability of different PARPi to inhibit the catalytic activity of PARP family members does not directly correlate with cytotoxic potential. All PARPi contain a benzamide moiety, which is a key feature for binding to PARP-1, but each inhibitor differs in size and flexibility, accounting for different affinities and trapping abilities. Recently, Zandarashvili et al.^40^ proposed a molecular mechanism for PARPi-induced PARP-1 trapping in a study that revealed that a panel of clinically approved PARPi drives diverse allosteric changes in PARP-1 at DNA breaks, leading to differential potency in trapping. Talazoparib and olaparib were both classified as neutral toward PARP-1 allostery, with low affinity for a DNA break; conversely, rucaparib, niraparib, and veliparib were classified as allosteric pro-release PARPi^40^. Nonetheless, using a fluorescence anisotropy DNA-binding assay, Murai et al.^6,47^ reported that talazoparib was ~100-fold more efficient at trapping than niraparib, olaparib, and rucaparib. Talazoparib was also found to have the most profound cytotoxic effects. Therefore, the allosteric effect of different PARPi on PARP-1 trapping requires further study to resolve these inconsistencies.

Another interesting feature of PARPi to consider is substrate selectivity and specificity. Although PARPi are promiscuous among different PARP family members at higher concentrations (veliparib and niraparib are most selective toward PARP-1 and PARP-2)^48^, a recent study by Antolin et al.^46^ used three computational in silico analyses to systematically uncover the molecular target profile of PARPi across the human kinome. This method predicted 58 potential interactions with kinases, only 10 of which were previously known. Furthermore, catalytic inhibition assays provided evidence that rucaparib was able to inhibit the activity of three kinases (CDK16, PIM3, and DYRK1B) and that niraparib inhibited two kinases (DYRK1A and DYRK1B), all at submicromolar IC_50_ values (Table 1). The authors speculated that all PARPi have some intrinsic capacity to inhibit other kinases more generally and that each inhibitor may have unique off-target kinase targets, which likely account for PARPi efficiencies and distinct clinical adverse side-effect patterns^46^. In addition, Knezevic et al.^49^ used an unbiased, mass spectrometry-based chemical proteomics approach to identify hexose-6-phosphate and deoxycytidine kinase as targets of rucaparib and niraparib, respectively. These studies stress the need to carefully consider the system-wide effects of inhibitors to predict their clinical benefit.

### PARPi maintenance monotherapies

Encouraging results in specific patient cohorts have been obtained for PARPi used as monotherapies. Olaparib was first approved in December 2014 as a maintenance monotherapy for women with recurrent ovarian cancer, who harbored germline *BRCA1/2* mutations after complete or partial response to multiple first-line platinum-based chemotherapies^50^, and olaparib and talazoparib have been approved as maintenance monotherapies in HER2-negative metastatic breast cancer with germline *BRCA1/2* mutations. Various studies have provided further evidence of PARPi efficacy as a monotherapy; e.g., olaparib was just as effective as pegylated liposomal doxorubicin (PLD) in a randomized Phase 2 study of ovarian cancer patients with mutated *BRCA1/2* (NCT00628251)^51^. In addition, olaparib had a higher objective response rate (ORR) compared to chemotherapy (paclitaxel, topotecan, gemcitabine, or PLD) (NCT02282020) in a Phase 3 study in patients with mutated *BRCA1/2* status and platinum-sensitive recurrent ovarian cancer^52^. Rucaparib provided the highest PFS and ORR in a *BRCA1/2*-mutated cohort compared to a non-*BRCA1/2*-mutated cohort with high or low genomic loss of heterozygosity (NCT01891344)^10^. Platinum-resistant ovarian cancers also exhibit a moderate response to rucaparib, better ORR and tumor response rate in patients with *BRCA* mutation treated with olaparib, and moderate ORR in patients with *BRCA* mutations treated with niraparib^52^. Clinical trials are currently underway to assess the use of various PARPi in maintenance therapy of recurrent platinum-treated ovarian and breast cancer, both as single agents in the therapeutic setting of adjuvant and standard-of-care chemotherapy for advanced diseases^53^.

### PARPi combination strategies

Various studies and clinical trials are aiming to decipher and predict the best combination approaches to further potentiate the efficacy of PARPi^14,54^. The rationale for combining PARPi with various cytotoxic chemotherapeutic agents is based on the link between HRD, DNA repair, and synthetic lethality described above. The increased genomic instability of HR-deficient tumor cells renders them highly sensitive to DNA-damaging platinum agents. The use of olaparib with a combination of carboplatin and paclitaxel in platinum-sensitive recurrent high-grade serous ovarian cancer has significantly improved PFS in patients^55^. Veliparib in combination with carboplatin and gemcitabine or paclitaxel is also beneficial for advanced ovarian cancer^56,57^. Many other DNA-damaging agents are being tested (cisplatin, PLD, topotecan, trabectedin, temozolomide)^54^. Similarly, PARPi are being tested in combination with ionizing radiation^54^.

Aside from DNA damage, PARPi trials are extensively testing combination therapies to exploit PARPi beyond *BRCA1/2*-mutated and platinum-sensitive cancers. Some of these combinations seek to overcome common mechanisms of acquired PARPi resistance (discussed below). These include combinations with anti-angiogenic agents (bevacizumab or cediranib), immune checkpoint blockers (tremelumimab, durvalumab, nivolumab, atezolizumab, pembrolizumab, avelumab, and TSR-042), DNA damage checkpoint inhibitors (AZD6738 and AZD1775), chaperone inhibitors (onalespib), kinase inhibitors for mTORC1/2 (AZD2014 and everolimus), AKT (AZD5363), MEK (cobimetinib), and PI3K (BKM120, BYL719, and copanlisib)^52,54^. These combination trials are being conducted in the neoadjuvant therapeutic settings and in advanced diseases^53^.

## Mechanisms of resistance to PARPi

Despite the clinical benefit of PARPi observed in patients harboring *BRCA1/2* mutations, the emergence of drug resistance remains a therapeutic challenge. Expanding our understanding of these mechanisms is necessary to counteract this resistance and improve therapeutic outcome.

### Restoration of HR capacity

**Fig. 3:**
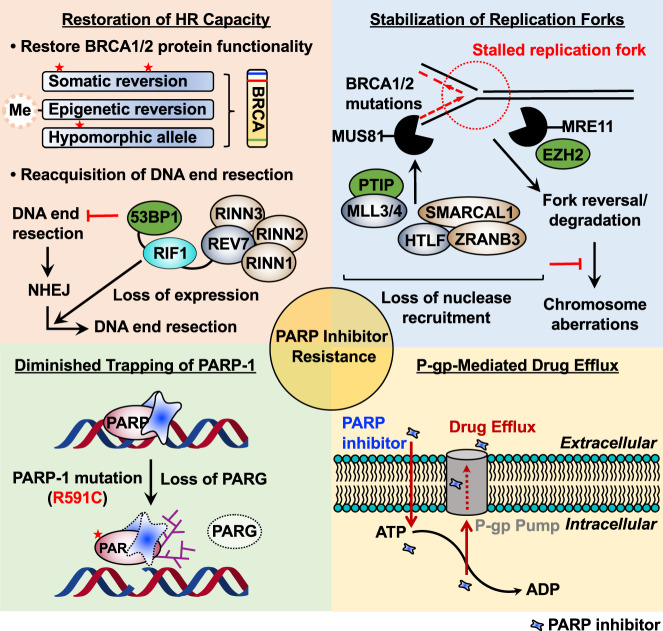
Mechanisms of resistance to PARP inhibitors. Cancer cells develop different resistance mechanisms that pose a significant obstacle to the prolonged use of PARPi. Several proposed molecular mechanisms of PARPi resistance, including restoration of HR capacity, stabilization of replication forks, diminished trapping of PARP-1, and P-gp-mediated drug efflux, are highlighted in four sections.

The most common acquired mechanism of resistance to PARPi appears to be through restoration of HR in HR-deficient tumors (Fig. 3, upper left panel)^20^. Genetic reversion mutations are somatic insertion/deletion mutations that cause a frameshift and restore the open reading frame, leading to expression of a functional protein and thereby rendering tumors partially HR proficient^19^. Somatic reversion mutations are highly clinically relevant for patients with *BRCA1/2*-mutated cancers, who are treated with platinum-based therapy, and they have been observed to occur in multiple other HR pathway genes, including *RAD51C*, *RAD51D*, and *PALB2*^58^. Loss of *BRCA1* promoter methylation is another mechanism that restores functional *BRCA1* expression to levels comparable to those in HR-proficient tumors^59^. Thus, both genetic and epigenetic mechanisms are at play.

Recent studies have provided preclinical evidence that truncated, but hypomorphic, *BRCA1* splice isoforms lacking exon 11 are able to form RAD51 and BRCA1 foci in response to ionizing radiation, indicating partial HR proficiency^60^. In addition, tumors carrying the *BRCA1*^*C61G*^ mutation, which disrupts the N-terminal RING domain, rapidly develop PARPi resistance due to residual activity in DNA damage responses^61^. Furthermore, HSP90 interacts with and stabilizes the C-terminal-truncated BRCA1 protein under PARPi selection pressure^62^. This stabilized mutant BRCA1 protein maintains partial function; it is able to promote RAD1 loading onto DNA, thereby conferring PARPi resistance.

Another mechanism for restoration of HR capacity is reacquisition of DNA end resection that promotes restarting of the HR pathway (Fig. 3, upper left panel). HR capacity can also be restored by loss of 53BP1 or end resection-associated factors^63^. As BRCA1 inhibits 53BP1 to initiate DSB repair, loss of BRCA1 prevents the release of 53BP1 from DNA ends and secures arrested HR repair. 53BP1 and resection-associated factors, including RIF1, REV7, and shieldin, are recruited to DSBs and block HR by limiting DNA end resection^63^. Loss of function of this pathway results in resection, leads to RAD51 recruitment in the absence of BRCA1, and restores HR function, rendering cells resistant to PARPi. Collectively, reversion mutations of HR pathway genes and reacquisition of DNA end resection cause restoration of HR capacity, conferring PARPi resistance.

### Stabilization of replication forks

In addition to somatic reversion of mutated *BRCA1/2* alleles, *BRCA1/2*-deficient cells have evolved the ability to use alternative mechanisms that inhibit DNA replication fork degradation by nucleases to stabilize the replication fork (Fig. 3, upper right panel)^64,65^. BRCA1 and BRCA2 are required for the protection of stalled replication forks caused by replication stress. In the absence of BRCA1/2, nucleases MRE11^64^ and MUS81^65^ attack stalled replication forks, leading to fork collapse and chromosomal aberrations. The methyltransferases EZH2 and PTIP play a key role in recruiting MUS81 and MRE11 to the stalled replication fork, respectively^66^. However, in *BRCA1/2*-deficient cells, EZH2 (BRCA2-deficient) and PTIP (BRCA1-deficient) activity is downregulated at the fork, reducing recruitment of nucleases and resulting in fork protection. In addition, fork reversal or remodeling by the chromatin remodelers SMARCAL1, ZRANB3, and HLTF has been shown to be required for MRE11-dependent degradation of replication forks^64^. Loss of these factors in *BRCA1/2*-deficient cells leads to fork head protection and results in PARPi resistance. PARPi are known to induce fork degradation of unprotected replication forks; therefore, increased stabilization of replication forks confers PARPi resistance^66,67^. Various strategies are used in *BRCA1/2*-mutated cells, as described above, to protect their replication forks, inducing PARPi resistance without restoring HR repair.

### Diminished trapping of PARP-1

Recent studies have proposed that inhibition of PARP trapping activity can confer PARPi resistance (Fig. 3, lower left panel)^60,68^. PARP trapping by PARPi leads to the accumulation of unrepaired SSBs and impaired progression of replication forks, inducing cell death^6,41^. The first clinical evidence of a functional link between PARP trapping and PARPi resistance was proposed by Pettitt et al.^60^. These researchers identified a PARP-1 mutation (R591C) commonly observed in PARPi-resistant patient tumor samples, which was associated with diminished trapping of PARP-1 on DNA, resulting in PARPi resistance. This finding suggests that PARP-1 mutations can decrease DNA trapping and induce PARPi resistance.

PARylation is reversed by PARG. Accordingly, PARG acts in parallel to PARPi by preventing PAR accumulation. In PARPi-treated cells, loss of PARG leads to accumulation of PAR, which reduces PARP-1 trapping on DNA by rescuing PARP-1-dependent DNA damage signaling, resulting in PARPi resistance^68^. Collectively, clinically relevant PARP-1 mutations and loss of PARG in PARPi-treated cells hinder the trapping of PARP-1 and confer PARPi resistance.

### P-gp-mediated drug efflux

*Abcb1a/b* genes encoding P-glycoprotein (P-gp) efflux pumps have been implicated as a common mechanism of chemotherapeutic resistance across different classes of drugs. Indeed, overexpression of P-gp efflux pumps by chromosomal translocations of *Abcb1a/b* genes decreases the efficiency of a variety of compounds by enhancing their extracellular translocation. Hence, overexpression of P-gp efflux pumps is also associated with PARPi resistance (Fig. 3, lower right panel)^69,70^. For example, overexpression of P-gp efflux pumps has been observed in a PARPi-resistant human cancer cell line, and cotreatment with the P-gp efflux pump inhibitor tariquidar resensitized the tumors to PARPi^69^. Regardless, clinical trials targeting P-gp efflux pumps in the context of PARPi resistance have not yet been reported^71^. Therefore, future clinical studies should address the fundamental mechanisms between increased expression of P-gp efflux pumps and PARPi resistance.

### Other proposed mechanisms of PARPi resistance

Other proposed mechanisms of PARPi resistance include the following.

1. Alterations in cell cycle control: Overexpression of cell cycle regulators such as cyclin-dependent kinase 12 (CDK12) and WEE1 have been found to promote PARPi resistance by restoring HR^72^. Induction of “BRCAness” is phenocopied by knockdown of CDK12, which leads to downregulation of DNA repair proteins, thereby conferring sensitivity to PARPi. Under PARPi treatment in HRD, WEE1 inhibition forces cells to enter mitosis without completing DNA synthesis and repair, resulting in the accumulation of DNA DSBs and promoting sensitivity to PARPi.
2. miRNA expression patterns: A number of studies have observed that altered miRNA expression patterns are associated with PARPi resistance^73–75^. For example, miR-622 induces PARPi resistance by directly inhibiting expression of NHEJ components (Ku70/80), enhancing the HR pathway^73^. In addition, antagonizing miR-182 increases BRCA1 protein levels and promotes HR and subsequent resistance to PARPi^74^.
3. Dysregulated signaling pathways: Aberrant regulation of multiple signaling pathways, such as MET, PI3K/AKT, and ATM/ATR, has been reported to be associated with PARPi resistance^76,77^. The receptor tyrosine kinase c-Met directly phosphorylates PARP-1, which leads to activation of PARP-1 enzymatic activity and reduces the binding affinity of PARPi, thereby conferring PARPi resistance^76^. In addition, treatment with PARPi causes upregulation of the PI3K/AKT pro-survival pathway, which is a key regulator of cell growth and proliferation^77^. Two serine/threonine kinases, ATM and ATR, are essential factors in the DNA damage response pathway, acting through the phosphorylation of histone H2A and recruitment of DNA repair complexes^75^. Therefore, upregulation of the ATM/ATR pathway confers resistance to PARPi through HR restoration and inhibition of these kinases may be an efficient method to overcome PARPi resistance.

## Conclusions and perspectives

Based on a number of clinical and mechanistic studies, PARPi clearly represent a major breakthrough as a therapeutic for various gynecological cancers. This is especially true for cancers with a mutated *BRCA1/2* status or HR-mediated DNA repair deficiency, but more excitingly, new data also suggest a clinical benefit irrespective of *BRCA1/2* mutations. A more in-depth understanding of the molecular mechanisms that underlie the action of PARPi, including trapping, will be important for strengthening the rationale to extend the use of PARPi to a broader array of patient populations. In addition, more detailed mechanistic studies will help to elucidate biomarkers that can predict response and help guide clinical decisions on the best use of combination therapies that will have the greatest synergistic effects in individual cancers. These much-needed molecular studies, along with the completion of many ongoing PARPi trials, will improve current standard-of-care treatment regimens by maximally exploiting the use of PARPi in the clinic. In addition, PARPi resistance mechanisms may be important for determining the therapeutic potential of PARPi, although many have not been found to be clinically relevant, except for *BRCA1/2* reversion mutations. Therefore, further mechanistic and clinical studies evaluating PARPi resistance will be required to reveal the relevance of different resistance mechanisms in the clinic.

## References

[1] Kim DS, Challa S, Jones A, Kraus WL (2020). PARPs and ADP-ribosylation in RNA biology: from RNA expression and processing to protein translation and proteostasis. Genes Dev..

[2] Kim MY, Zhang T, Kraus WL (2005). Poly(ADP-ribosyl)ation by PARP-1: ‘PAR-laying’ NAD+ into a nuclear signal. Genes Dev..

[3] Ossovskaya V, Koo IC, Kaldjian EP, Alvares C, Sherman BM (2010). Upregulation of poly (ADP-ribose) polymerase-1 (PARP1) in triple-negative breast cancer and other primary human tumor types. Genes Cancer.

[4] Zaremba T (2009). Poly(ADP-ribose) polymerase-1 polymorphisms, expression and activity in selected human tumour cell lines. Br. J. Cancer.

[5] Farmer H (2005). Targeting the DNA repair defect in BRCA mutant cells as a therapeutic strategy. Nature.

[6] Murai J (2012). Trapping of PARP1 and PARP2 by clinical PARP inhibitors. Cancer Res..

[7] Pommier Y, O’Connor MJ, de Bono J (2016). Laying a trap to kill cancer cells: PARP inhibitors and their mechanisms of action. Sci. Transl. Med..

[8] Bryant HE (2009). PARP is activated at stalled forks to mediate Mre11-dependent replication restart and recombination. EMBO J..

[9] Yang YG, Cortes U, Patnaik S, Jasin M, Wang ZQ (2004). Ablation of PARP-1 does not interfere with the repair of DNA double-strand breaks, but compromises the reactivation of stalled replication forks. Oncogene.

[10] Swisher EM (2017). Rucaparib in relapsed, platinum-sensitive high-grade ovarian carcinoma (ARIEL2 Part 1): an international, multicentre, open-label, phase 2 trial. Lancet Oncol..

[11] Ledermann J (2014). Olaparib maintenance therapy in patients with platinum-sensitive relapsed serous ovarian cancer: a preplanned retrospective analysis of outcomes by BRCA status in a randomised phase 2 trial. Lancet Oncol..

[12] Moore K (2018). Maintenance olaparib in patients with newly diagnosed advanced ovarian cancer. N. Engl. J. Med.

[13] Pilie PG, Gay CM, Byers LA, O’Connor MJ, Yap TA (2019). PARP inhibitors: extending benefit beyond BRCA-mutant cancers. Clin. Cancer Res..

[14] Slade D (2020). PARP and PARG inhibitors in cancer treatment. Genes Dev..

[15] Mirza MR (2016). Niraparib maintenance therapy in platinum-sensitive, recurrent ovarian cancer. N. Engl. J. Med..

[16] Kim DS (2019). Activation of PARP-1 by snoRNAs controls ribosome biogenesis and cell growth via the RNA helicase DDX21. Mol. Cell.

[17] Keung, M. Y., Wu, Y., Badar, F. & Vadgama, J. V. Response of breast cancer cells to PARP inhibitors is independent of BRCA status. *J. Clin. Med.* **9**, 10.3390/jcm9040940 (2020). 10.3390/jcm9040940 PMC7231148 32235451

[18] Jaspers JE (2013). Loss of 53BP1 causes PARP inhibitor resistance in Brca1-mutated mouse mammary tumors. Cancer Discov..

[19] Edwards SL (2008). Resistance to therapy caused by intragenic deletion in BRCA2. Nature.

[20] Noordermeer SM, van Attikum H (2019). PARP inhibitor resistance: a tug-of-war in BRCA-mutated cells. Trends Cell Biol..

[21] Chambon P, Weill JD, Mandel P (1963). Nicotinamide mononucleotide activation of new DNA-dependent polyadenylic acid synthesizing nuclear enzyme. Biochem. Biophys. Res. Commun..

[22] Okazaki H, Niedergang C, Mandel P (1980). Adenosine diphosphate ribosylation of histone H1 by purified calf thymus polyadenosine diphosphate ribose polymerase. Biochimie.

[23] Ferro AM, Olivera BM (1982). Poly(ADP-ribosylation) in vitro. Reaction parameters and enzyme mechanism. J. Biol. Chem..

[24] de Murcia G, Jongstra-Bilen J, Ittel ME, Mandel P, Delain E (1983). Poly(ADP-ribose) polymerase auto-modification and interaction with DNA: electron microscopic visualization. EMBO J..

[25] Alvarez-Gonzalez R, Jacobson MK (1987). Characterization of polymers of adenosine diphosphate ribose generated in vitro and in vivo. Biochemistry.

[26] Nolan NL, Butt TR, Wong M, Lambrianidou A, Smulson ME (1980). Characterization of poly(ADP-ribose)-histone H1 complex formation in purified polynucleosomes and chromatin. Eur. J. Biochem..

[27] de Murcia G (1986). Modulation of chromatin superstructure induced by poly(ADP-ribose) synthesis and degradation. J. Biol. Chem..

[28] Skidmore CJ (1979). The involvement of poly(ADP-ribose) polymerase in the degradation of NAD caused by gamma-radiation and N-methyl-N-nitrosourea. Eur. J. Biochem..

[29] Satoh MS, Lindahl T (1992). Role of poly(ADP-ribose) formation in DNA repair. Nature.

[30] Ruf A, Mennissier de Murcia J, de Murcia G, Schulz GE (1996). Structure of the catalytic fragment of poly(AD-ribose) polymerase from chicken. Proc. Natl Acad. Sci. USA.

[31] Fong PC (2009). Inhibition of poly(ADP-ribose) polymerase in tumors from BRCA mutation carriers. N. Engl. J. Med..

[32] Weil MK, Chen AP (2011). PARP inhibitor treatment in ovarian and breast cancer. Curr. Probl. Cancer.

[33] Ray Chaudhuri A, Nussenzweig A (2017). The multifaceted roles of PARP1 in DNA repair and chromatin remodelling. Nat. Rev. Mol. Cell Biol..

[34] Lord CJ, Ashworth A (2017). PARP inhibitors: synthetic lethality in the clinic. Science.

[35] Michel B (2001). Rescue of arrested replication forks by homologous recombination. Proc. Natl Acad. Sci. USA.

[36] Chen CC, Feng W, Lim PX, Kass EM, Jasin M (2018). Homology-directed repair and the role of BRCA1, BRCA2, and related proteins in genome integrity and cancer. Annu. Rev. Cancer Biol..

[37] van Wietmarschen N, Nussenzweig A (2018). Mechanism for synthetic lethality in BRCA-deficient cancers: no longer lagging behind. Mol. Cell.

[38] Bryant HE (2005). Specific killing of BRCA2-deficient tumours with inhibitors of poly(ADP-ribose) polymerase. Nature.

[39] Maya-Mendoza A (2018). High speed of fork progression induces DNA replication stress and genomic instability. Nature.

[40] Zandarashvili, L. et al. Structural basis for allosteric PARP-1 retention on DNA breaks. *Science* **368**, 10.1126/science.aax6367 (2020). 10.1126/science.aax6367 PMC7347020 32241924

[41] Murai J, Pommier Y (2019). PARP trapping beyond homologous recombination and platinum sensitivity in cancers. Annu. Rev. Cancer Biol..

[42] Ruggero D, Pandolfi PP (2003). Does the ribosome translate cancer?. Nat. Rev. Cancer.

[43] Xu H (2017). CX-5461 is a DNA G-quadruplex stabilizer with selective lethality in BRCA1/2 deficient tumours. Nat. Commun..

[44] Peltonen K (2014). A targeting modality for destruction of RNA polymerase I that possesses anticancer activity. Cancer Cell.

[45] Huang D, Kim DS, Kraus WL (2020). Specific binding of snoRNAs to PARP-1 promotes NAD(+)-dependent catalytic activation. Biochemistry.

[46] Antolin AA (2020). The kinase polypharmacology landscape of clinical PARP inhibitors. Sci. Rep..

[47] Murai J (2014). Stereospecific PARP trapping by BMN 673 and comparison with olaparib and rucaparib. Mol. Cancer Ther..

[48] Thorsell AG (2017). Structural basis for potency and promiscuity in poly(ADP-ribose) polymerase (PARP) and tankyrase inhibitors. J. Med. Chem..

[49] Knezevic CE (2016). Proteome-wide profiling of clinical PARP inhibitors reveals compound-specific secondary targets. Cell Chem. Biol..

[50] Kaufman B (2015). Olaparib monotherapy in patients with advanced cancer and a germline BRCA1/2 mutation. J. Clin. Oncol..

[51] Kaye SB (2012). Phase II, open-label, randomized, multicenter study comparing the efficacy and safety of olaparib, a poly (ADP-ribose) polymerase inhibitor, and pegylated liposomal doxorubicin in patients with BRCA1 or BRCA2 mutations and recurrent ovarian cancer. J. Clin. Oncol..

[52] Lightfoot M, Montemorano L, Bixel K (2020). PARP inhibitors in gynecologic cancers: what is the next big development?. Curr. Oncol. Rep..

[53] Cerrato A, Morra F, Celetti A (2016). Use of poly ADP-ribose polymerase [PARP] inhibitors in cancer cells bearing DDR defects: the rationale for their inclusion in the clinic. J. Exp. Clin. Cancer Res..

[54] Jiang X, Li W, Li X, Bai H, Zhang Z (2019). Current status and future prospects of PARP inhibitor clinical trials in ovarian cancer. Cancer Manag. Res..

[55] Parmar MK (2003). Paclitaxel plus platinum-based chemotherapy versus conventional platinum-based chemotherapy in women with relapsed ovarian cancer: the ICON4/AGO-OVAR-2.2 trial. Lancet.

[56] Gray HJ (2018). Phase I combination study of the PARP inhibitor veliparib plus carboplatin and gemcitabine in patients with advanced ovarian cancer and other solid malignancies. Gynecol. Oncol..

[57] Nishio S (2017). Phase 1 study of veliparib with carboplatin and weekly paclitaxel in Japanese patients with newly diagnosed ovarian cancer. Cancer Sci..

[58] Kondrashova O (2017). Secondary somatic mutations restoring RAD51C and RAD51D associated with acquired resistance to the PARP inhibitor Rucaparib in high-grade ovarian carcinoma. Cancer Discov..

[59] Ter Brugge, P. et al. Mechanisms of therapy resistance in patient-derived xenograft models of BRCA1-deficient breast cancer. *J. Natl Cancer Inst.* **108**, 10.1093/jnci/djw148 (2016). 10.1093/jnci/djw148 27381626

[60] Pettitt SJ (2018). Genome-wide and high-density CRISPR-Cas9 screens identify point mutations in PARP1 causing PARP inhibitor resistance. Nat. Commun..

[61] Drost R (2011). BRCA1 RING function is essential for tumor suppression but dispensable for therapy resistance. Cancer Cell.

[62] Johnson N (2013). Stabilization of mutant BRCA1 protein confers PARP inhibitor and platinum resistance. Proc. Natl Acad. Sci. USA.

[63] Bunting SF (2010). 53BP1 inhibits homologous recombination in Brca1-deficient cells by blocking resection of DNA breaks. Cell.

[64] Taglialatela A (2017). Restoration of replication fork stability in BRCA1- and BRCA2-deficient cells by inactivation of SNF2-family fork remodelers. Mol. Cell.

[65] Rondinelli B (2017). EZH2 promotes degradation of stalled replication forks by recruiting MUS81 through histone H3 trimethylation. Nat. Cell Biol..

[66] Ray Chaudhuri A (2016). Replication fork stability confers chemoresistance in BRCA-deficient cells. Nature.

[67] Liao, H., Ji, F., Helleday, T. & Ying, S. Mechanisms for stalled replication fork stabilization: new targets for synthetic lethality strategies in cancer treatments. *EMBO Rep.* **19**, 10.15252/embr.201846263 (2018). 10.15252/embr.201846263 PMC6123652 30108055

[68] Gogola E (2019). Selective loss of PARG restores PARylation and counteracts PARP inhibitor-mediated synthetic lethality. Cancer Cell.

[69] Rottenberg S (2008). High sensitivity of BRCA1-deficient mammary tumors to the PARP inhibitor AZD2281 alone and in combination with platinum drugs. Proc. Natl Acad. Sci. USA.

[70] Jaspers JE (2015). BRCA2-deficient sarcomatoid mammary tumors exhibit multidrug resistance. Cancer Res..

[71] Bitler BG, Watson ZL, Wheeler LJ, Behbakht K (2017). PARP inhibitors: clinical utility and possibilities of overcoming resistance. Gynecol. Oncol..

[72] Bajrami I (2014). Genome-wide profiling of genetic synthetic lethality identifies CDK12 as a novel determinant of PARP1/2 inhibitor sensitivity. Cancer Res..

[73] Choi YE (2016). Platinum and PARP inhibitor resistance due to overexpression of microRNA-622 in BRCA1-mutant ovarian cancer. Cell Rep..

[74] Moskwa P (2011). miR-182-mediated downregulation of BRCA1 impacts DNA repair and sensitivity to PARP inhibitors. Mol. Cell.

[75] Teng PN (2015). Pharmacologic inhibition of ATR and ATM offers clinically important distinctions to enhancing platinum or radiation response in ovarian, endometrial, and cervical cancer cells. Gynecol. Oncol..

[76] Du Y (2016). Blocking c-Met-mediated PARP1 phosphorylation enhances anti-tumor effects of PARP inhibitors. Nat. Med..

[77] Tapodi A (2005). Pivotal role of Akt activation in mitochondrial protection and cell survival by poly(ADP-ribose)polymerase-1 inhibition in oxidative stress. J. Biol. Chem..

